# Hyperviscosity‐Related Ischemic Stroke in an Adolescent With Unrepaired Tetralogy of Fallot: A Case Report From a Resource‐Limited Setting

**DOI:** 10.1002/ccr3.72415

**Published:** 2026-04-03

**Authors:** Ayenew A. Wolie, Chernet T. Mengistie, Biruk T. Mengistie, Elleni D. Mulatu, Rahel N. Teferi, Birhanu T. Andeta, Gedefaw T. Minwagaw

**Affiliations:** ^1^ Department of Emergency Medicine and Critical Care School of Medicine, College of Health Sciences, Addis Ababa University Addis Ababa Ethiopia; ^2^ School of Medicine, College of Health Sciences, Addis Ababa University Addis Ababa Ethiopia; ^3^ Department of Neurology, School of Medicine College of Health Sciences, Addis Ababa University Addis Ababa Ethiopia

**Keywords:** cyanotic congenital heart disease, ischemic stroke, phlebotomy, secondary polycythaemia, tetralogy of Fallot

## Abstract

Tetralogy of Fallot (TOF) is the most common cyanotic congenital heart disease. Chronic hypoxemia in uncorrected TOF causes compensatory erythrocytosis and hyperviscosity, predisposing to cerebrovascular complications. Stroke in this context is rare but clinically significant, especially in resource‐limited settings where access to imaging and surgical correction is restricted. We report a 17‐year‐old male with unrepaired TOF and secondary polycythaemia who presented with recurrent headaches and dizziness following repeated phlebotomy. He subsequently developed acute right‐sided weakness and aphasia. Brain MRI revealed an acute ischemic infarction in the left basal ganglia and insular region. Echocardiography confirmed TOF anatomy without thrombus or new shunt changes. The patient was treated with supportive measures, antiplatelet therapy, and hydration; further phlebotomy was withheld. Neurologic improvement occurred with rehabilitation, though residual weakness persisted at discharge. This case highlights ischemic stroke as a rare but serious manifestation of hyperviscosity in cyanotic congenital heart disease. In resource‐limited settings, pragmatic diagnostic strategies and cautious hematologic management are vital. Early recognition of neurologic symptoms, avoidance of excessive phlebotomy, and timely referral for definitive TOF repair are essential to prevent recurrence and improve outcomes.

## Key Clinical Message

In resource‐limited settings, therapeutic phlebotomy for secondary erythrocytosis in uncorrected Tetralogy of Fallot requires caution. Excessive or poorly monitored venesection may increase viscosity and precipitate ischemic complications. Hydration status, hematocrit trends, and clinical condition should guide therapy; early definitive repair prevents recurrence.

## 1 Introduction

Tetralogy of Fallot (TOF) is a conotruncal congenital heart defect comprising four anatomical abnormalities (ventricular septal defect, right ventricular outflow tract obstruction, right ventricular hypertrophy, and an overriding aorta) [1]. It is the prototypical cyanotic heart lesion. Improvements in pediatric cardiology have increased survival, with over 90% of children with congenital heart disease (CHD) now reaching adulthood [2]. Nevertheless, TOF remains a major cyanotic CHD encountered in clinical practice, and unrepaired cases persist in settings with limited resources [1, 3, 4]. Chronic hypoxemia from right‐to‐left shunting in TOF stimulates erythropoietin production and causes compensatory secondary erythrocytosis [5]. This adaptive increase in red cell mass raises arterial oxygen content but dramatically increases blood viscosity, impairing microvascular perfusion [5, 6]. The resulting hyperviscosity syndrome can lead to symptoms such as headache, dizziness, visual disturbances, fatigue, and paresthesia [5, 7]. Importantly, hyperviscosity predisposes to vascular thrombosis; overt thrombotic events in cyanotic CHD (including ischemic stroke and myocardial infarction) are well‐documented [5].

Patients with CHD, especially complex or cyanotic lesions, have markedly elevated stroke risk [8]. In adults with CHD, stroke incidence is several‐fold higher than in the general population, and cyanotic physiology carries the greatest risk [8, 9, 10]. Stroke in CHD can occur at a younger age than a typical atherosclerotic stroke, and may present as the first manifestation of an unrecognized cardiac defect [8, 9]. Standard evaluation of Tetralogy of Fallot (TOF) and its neurologic complications relies primarily on echocardiography to define intracardiac anatomy, supplemented by advanced cross‐sectional imaging (MRI/CT) when needed to characterize cerebral injury or extracardiac pathology [2, 11, 12]. Definitive therapy for TOF is complete surgical repair, usually performed in infancy or early childhood. After repair, long‐term transplant‐free survival is excellent (over 94% at 25 years) [11, 13]. By contrast, uncorrected TOF leads to progressive cyanosis, heart failure, and early mortality if not treated [11]. Medical treatment of Tetralogy of Fallot focuses on preventing complications. Symptomatic hyperviscosity is managed with proper hydration and, if necessary, careful phlebotomy [5, 6, 7]. In summary, TOF's pathophysiology of chronic cyanosis with secondary polycythemia underlies the risk of hyperviscosity and stroke, and modern management emphasizes early correction and avoidance of complications [2, 5].

This case describes an adolescent with uncorrected Tetralogy of Fallot and severe secondary polycythemia who developed an acute ischemic stroke shortly after therapeutic phlebotomy, in a resource‐limited setting. It emphasizes the importance of early surgical correction, careful reduction of hematocrit, proper fluid replacement, and individualized antithrombotic treatment in patients with cyanotic congenital heart disease.

## 2 Clinical History/Examination

A 17‐year‐old male with known, uncorrected Tetralogy of Fallot (TOF) and a history of secondary polycythaemia, presented to the emergency department with recurrent headaches, dizziness, and exertional shortness of breath. His chronic medications included furosemide, spironolactone, metoprolol, and sodium valproate for a seizure disorder. He had been receiving intermittent therapeutic phlebotomy for symptomatic erythrocytosis. Definitive surgical correction had not been performed because of limited access to specialized cardiac surgical services.

On arrival, his vital signs were blood pressure 88–90/60–68 mmHg, pulse 90–96 beats/min, respiratory rate 22 breaths/min, and oxygen saturation 85% on room air. A therapeutic phlebotomy was performed, and he was discharged. Five days later, he returned with recurrence of the same symptoms and was kept in the emergency unit for evaluation and planned symptomatic phlebotomy pending laboratory results. While awaiting investigations, he suddenly developed right‐sided weakness and facial deviation to the left, without loss of consciousness, convulsions, fever, or bleeding tendencies.

On examination, he was alert and oriented (GCS 15/15). There was right‐sided supranuclear facial palsy and expressive aphasia, with preserved comprehension but impaired fluency, naming, reading, and writing. Muscle strength was 0/5 in the right upper limb and 2/5 in the right lower limb, with increased tone and brisk deep tendon reflexes on the right side (patellar reflex +3), compared to +2 on the left. The right plantar response was extensor, and visual fields were grossly intact.

## 3 Differential Diagnosis, Investigations, and Treatment

**FIGURE 1:**
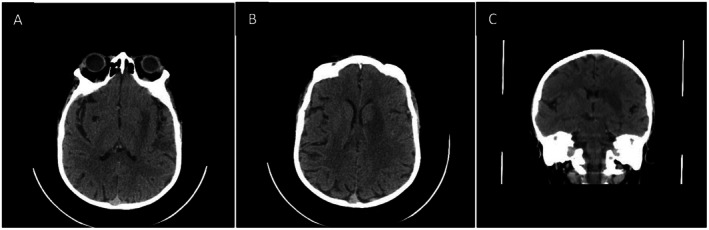
Non‐contrast brain CT (A–C). Axial non‐contrast CT images showing no acute hemorrhage. Subtle hypodensity is noted in the left basal ganglia and insular region (arrows), raising suspicion for early ischemic change.

**FIGURE 2:**
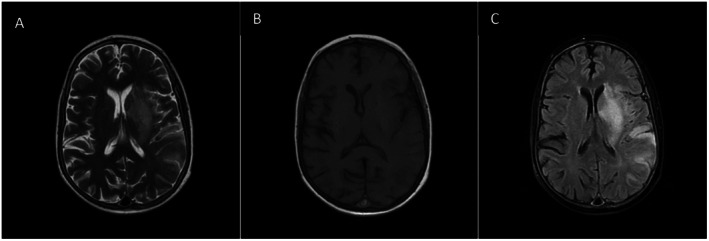
MRI Axial sequences (A–C). (A) Axial T2‐weighted image showing hyperintense signal in the left basal ganglia and insular cortex consistent with edema from acute infarction. (B) Axial T1‐weighted image demonstrating corresponding hypointensity in the same region. (C) Axial FLAIR sequence delineating the extent of the infarction involving the left corpus striatum, insula, and adjacent temporal–parietal cortex. Arrows indicate the affected area.

**FIGURE 3:**
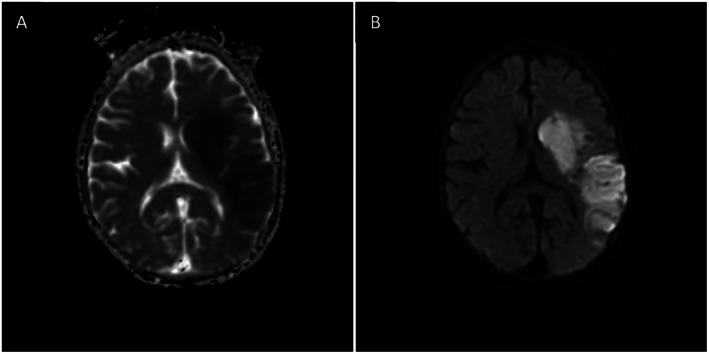
Diffusion‐weighted MRI (A, B). (A) DWI showing marked diffusion restriction in the left corpus striatum and insular cortex (arrowheads). (B) Corresponding ADC map demonstrating low signal in the same regions, confirming true diffusion restriction and acute infarction.

Laboratory investigations revealed a hematocrit of 62.3%, red blood cell count of 7.79 × 10^6^/μL, mean corpuscular volume of 80 fL, and red cell distribution width of 26.5%. Iron studies, renal function, and electrolytes were within normal limits. Lipid profile was unremarkable except for low HDL cholesterol (34 mg/dL). Non‐contrast brain CT showed no acute hemorrhage but subtle hypodensity in the left basal ganglia and insular region (Figure 1A–C). Subsequent MRI of the brain demonstrated acute ischemic changes involving the left corpus striatum, insular region, and adjacent temporal and parietal cortices (Figure 2A–C), showing hyperintensity on diffusion‐weighted imaging (DWI) and corresponding low signal on the apparent diffusion coefficient (ADC) map (Figure 3A,B). MR venography excluded cerebral venous thrombosis. Arterial vascular imaging (MRA) was not performed. An echocardiogram performed during admission demonstrated the typical TOF anatomy without any new abnormalities, intracardiac thrombus, or changes in shunt physiology.

Given the temporal relationship between recent phlebotomy and symptom onset, along with markedly elevated hematocrit and focal left hemispheric infarction, the diagnosis of acute ischemic stroke secondary to polycythaemia in the setting of uncorrected TOF was made. Phlebotomy was withheld, and he was managed with multidisciplinary input. Aspirin (81 mg daily), atorvastatin (40 mg daily), and subcutaneous unfractionated heparin (5000 IU twice‐daily) were initiated. Diuretics were temporarily withheld to prevent further volume depletion, and sodium valproate was continued. Supportive care with oxygen, hydration, and close neurological monitoring was provided.

## 4 Outcome and Follow‐Up

The patient showed gradual improvement over the following week. At discharge, right upper limb power was 1/5 and right lower limb power 3/5. Hematocrit remained elevated (62.3%). He was discharged on aspirin and atorvastatin, with plans for multidisciplinary follow‐up and further evaluation, including repeat echocardiography, iron studies, and hematologic assessment.

## 5 Discussion

Stroke due to hyperviscosity in untreated TOF is uncommon but has been reported in case series and reports of congenital cyanotic heart disease [5, 8, 9]. Mechanisms include sluggish cerebral flow from increased viscosity, in situ thrombosis of small vessels, and paradoxical embolism through intracardiac shunts [7]. In polycythemic TOF, focal ischemic deficits often occur in watershed or deep structures, but can involve any vascular territory [9, 14]. In our patient, marked erythrocytosis (Hct 62%) likely precipitated cerebral infarction despite initial phlebotomy. Observational data suggest that stroke in cyanotic CHD may present at young ages and in patients not yet diagnosed with cardiac disease [8].

Definitive diagnosis of acute ischemic stroke in adolescents is best achieved with brain MRI including diffusion‐weighted imaging (DWI), which is significantly more sensitive than non‐contrast CT for detecting early infarction and delineating viable tissue that guides management [12, 15]. When venous infarction or atypical lesion distribution is suspected, magnetic resonance venography (MRV) complements MRI by detecting cerebral venous thrombosis, which is often missed on CT [15]. Cardiac evaluation should begin with transthoracic echocardiography (TTE). Subsequently, when a cardioembolic source or complex anatomy is suspected, it should be followed by transesophageal echocardiography (TEE) owing to its superior visualization of left‐sided thrombi and aortic pathology [16, 17]. Detection of right‐to‐left shunts, relevant in cyanotic heart disease and paradoxical embolism, is optimally performed with contrast transcranial Doppler (TCD), a sensitive, non‐invasive screening tool that can guide further TEE assessment [17]. In settings where urgent advanced vascular imaging may be constrained or delayed, a pragmatic combination of CT to exclude hemorrhage, bedside echocardiography, focused hematologic testing, and multidisciplinary evaluation offers a feasible approach while recognizing diagnostic limitations [1, 12].

Management of erythrocytosis in cyanotic CHD requires balance: Guidelines recommend therapeutic phlebotomy only for symptomatic hyperviscosity (e.g., severe headache, visual changes), not routine venesection. This is because excessive phlebotomy can deplete iron stores and worsen outcomes [5, 7, 13]. Iron‐deficient erythropoiesis produces microcytic, rigid red cells that paradoxically increase blood viscosity and thrombosis risk [5, 7]. In this case, the patient had already been phlebotomized, and further phlebotomy was deferred to avoid worsening iron depletion. Although causality cannot be proven from a single case, recent therapeutic phlebotomy could plausibly have contributed by causing short‐term reductions in circulating volume and cardiac output with transient cerebral hypoperfusion, and by promoting iron‐restricted erythropoiesis with microcytosis and reactive thrombocytosis that increase thrombotic risk [18, 19]. Instead, supportive measures (oxygen, intravenous fluids) were used to improve rheology and cerebral perfusion [5, 13].

Anti‐thrombotic therapy in cyanotic CHD is empiric; many experts administer low‐dose aspirin in secondary erythrocytosis, analogous to polycythemia vera management, although data are limited [14, 20]. We therefore initiated antiplatelet therapy (aspirin, later clopidogrel) for presumed in situ thrombosis [20]. Selected cases of cyanotic‐CHD‐related stroke have been treated with thrombolysis or anticoagulation, but such interventions require individualized, multidisciplinary decision‐making because of altered hemostasis and bleeding risk in this population [14, 21]. Finally, definitive correction of the underlying cardiac lesion (complete TOF repair) remains the long‐term solution because normalization of oxygenation prevents recurrent erythrocytosis and its complications [11, 13]. Our patient, surviving to adolescence with unoperated TOF, remains at risk for future events until pulmonary blood flow can be established.

Outcomes in hyperviscosity stroke depend on prompt recognition and management of both the neurologic event and the underlying cause [5, 8]. Our patient demonstrated partial neurologic recovery with rehabilitation, a pattern described in individual case reports and small series of stroke complicating cyanotic congenital heart disease [14, 21]. Published data on stroke outcomes in cyanotic CHD remain limited. However, registry and cohort studies indicate that some patients achieve functional improvement with early intervention, while long‐term morbidity varies by lesion complexity and access to care [8, 9, 11]. That said, recurrent cerebrovascular events remain a concern if the primary lesion is not corrected [2, 10]. This case also highlights the diagnostic challenge: initial hyperviscosity symptoms (headache, dizziness) preceded focal signs, which might be dismissed or misattributed in a young patient without known stroke risk [5, 8]. High clinical suspicion and early neuroimaging are therefore crucial in cyanotic patients with neurologic complaints [12, 15].

In summary, this adolescent's ischemic stroke highlights the rare but serious neurologic complication of secondary erythrocytosis in TOF [5, 13]. Clinicians should recognize that therapeutic phlebotomy has a limited preventive effect and may paradoxically worsen hyperviscosity if overused [5, 7]. Optimal care involves a nuanced balance of hydration, judicious phlebotomy for symptoms, antithrombotic measures, and planning for definitive cardiac repair [6, 11, 14]. This approach is particularly important in resource‐limited settings where delays in definitive surgical care increase the risk of recurrent events and long‐term morbidity [1, 4].

## 6 Conclusion

This case illustrates a rare presentation of ischemic stroke in an adolescent with unrepaired Tetralogy of Fallot. It emphasizes the pathophysiologic role of chronic hypoxemia and polycythemia in precipitating cerebral hyperviscosity and thrombosis. Key learning points include recognition that neurological symptoms (headache, visual changes, and focal deficits) in cyanotic CHD may herald stroke, and that management must balance symptomatic hyperviscosity relief (hydration, selective phlebotomy) against the risk of iron depletion. Definitive repair of TOF is the ultimate preventive measure. This report highlights the importance of considering stroke in young patients with cyanotic heart disease and of promptly addressing both the neurologic event and its underlying cause to improve outcomes.

## Author Contributions

**Ayenew A. Wolie:** conceptualization, visualization, writing – original draft. **Chernet T. Mengistie:** conceptualization, data curation, writing – review and editing. **Biruk T. Mengistie:** data curation, visualization, writing – review and editing. **Elleni D. Mulatu:** resources, writing – original draft. **Rahel N. Teferi:** data curation, investigation. **Birhanu T. Andeta:** resources, supervision. **Gedefaw T. Minwagaw:** investigation, supervision.

## Funding

This research did not receive any specific grant from funding agencies in the public, commercial, or not‐for‐profit sectors.

## Ethics Statement

IRB review and approval were waived for this case report.

## Consent

Written informed consent for publication of the clinical details and accompanying images was obtained from the patient. The signed written informed consent form is held by the corresponding author and is available for review by the Editor upon request.

## Conflicts of Interest

The authors declare no conflicts of interest.

## Data Availability

The data underlying the results presented in this work are available within the manuscript.
